# Existential Threat: Uncovering Implicit Affect in Response to Terror Reminders in Soldiers

**DOI:** 10.3389/fpsyg.2021.585854

**Published:** 2021-06-04

**Authors:** Markus Quirin, Farhood Malekzad, Miguel Kazén, Udo Luckey, Hugo Kehr

**Affiliations:** ^1^Technical University of Munich, Private Hochschule Göttingen, Bavaria, Germany; ^2^Department of Psychology, Private Hochschule Göttingen, Göttingen, Germany; ^3^Institute of Psychology, Osnabrueck University, Osnabrück, Germany

**Keywords:** implicit affect, IPANAT, fear of terrorism, Afghanistan crisis, death awareness

## Abstract

Psychological science has a hard time assessing affective processes of the individuals that they may not recognize or do not like to report on. Here, the authors used the Implicit Positive and Negative Affect Test (IPANAT; Quirin et al., 2009) to investigate whether reminders of an existential threat induce unpleasant implicit affect in soldiers waiting for their deployment to a country with high levels of terrorist threat, Afghanistan. As expected, relative to reminding participants of a television evening, implicit negative affect was higher and implicit positive affect was lower after reminding participants of terror acts performed in different cities. No significant effects were found in self-reports of negative or positive affect. Our findings suggest that reminders of existential threat can elicit implicit negative affect that individuals may not report on explicitly and thus, validate the IPANAT as an easily applicable measure in emotional contexts.

## Introduction

Direct measures of experienced emotions may not reliably capture all relevant affective processes as cognitive and motivational factors bias reports of the individuals about them (cf. Robinson and Clore, 2002). As such, whenever such biasing factors are likely to occur, it is useful to assess affect by methods that circumvent subjective self-reports. This can particularly be the case when existential threats (e.g., the deployment of a soldier to a hazardous crisis area) are unavoidable and need to be dealt with in a way that everyday functioning can be maintained. The present experiment aimed to investigate whether an existential threat, in terms of reminders of terror acts, induces unpleasant affect at an implicit level in German soldiers who expected to be deployed to Afghanistan toward the end of the neo-Taliban Insurgency in 2007.

### Implicit Affect

Implicit affect may be defined as the automatic activation of cognitive associations of affective experiences (Quirin et al., 2009). Although several procedures, which indirectly assess attitudes, have been developed in the last two decades (e.g., Greenwald et al., 1998), indirect measures of affect have been largely missing. Quirin et al. (2009) remedied this situation by developing the IPANAT, an affect measure based on the judgment of artificial, meaningless words. The IPANAT is economically and easy to apply in a paper-pencil form and does not necessitate an elaborate application or score computation. Specifically, participants are asked to judge how much artificial words (e.g., TALEP) sound like several affective adjectives (see also Quirin and Bode, 2014, for a review of research using the IPANAT; Quirin et al., 2018, for the usability of the test in nine languages).

The IPANAT has been validated using questionnaire, priming, and endocrine, as well as (neuro)physiological data (for overviews, see Quirin et al., 2009; Weil et al., 2019). For example, research has provided evidence for the validity of the IPANAT that also measures state variance. For example, Quirin et al. (2009) provide criterion-based validity of the IPANAT by demonstrating that correlations between implicit affect and explicit affect are higher under spontaneous responding conditions than in the conditions under which participants reflectively respond to explicit affect scales. Their findings suggest that the IPANAT is a reliable and valid measure for affective processes with an economical application procedure. Moreover, implicit affect measured by the IPANAT was correlated with other indirect affect or stress indicators (such as cortisol or blood pressure), whereas direct measures of affect were uncorrelated (Quirin et al., 2009, 2011; Brosschot et al., 2014; Kazén et al., 2014). Quirin et al. (2009) found that the circadian increase in cortisol concentration correlates negatively with implicit positive affect while increases in cortisol concentration in response to acute stress correlate positively with implicit negative affect. At the same time, direct measures of affect were not significantly related to cortisol levels or changes. The IPANAT has also successfully been applied and validated in research on terror management (Quirin et al., 2014), adult attachment (Selcuk et al., 2012), mere exposure (Hicks and King, 2011), and individual differences in emotional awareness (Lichev et al., 2014).

### Affective Reactions in Response to Existential Threat

An existential threat has been shown to elicit defensive reactions that have been interpreted as mechanisms to ward off existential anxiety (Pyszczynski et al., 1999, 2003a). Existential threat also challenges the sense of control of the individual (Fritsche et al., 2008), induces uncertainty (McGregor et al., 2010), and is perceived as a violation of meaning (Proulx and Inzlicht, 2012). Yet, existential threat usually does *not* engender changes in self-reported affect, for example, in the form of increased anxiety or general negative affect ratings (for a meta-analysis, see Burke et al., 2010; but see Lambert et al., 2010).

Most empirical research on existential threat has so far been conducted under the framework of Terror Management Theory (TMT; Rosenblatt et al., 1989; Greenberg et al., 1990). TMT is founded on the assumption that humans first employ suppression or rationalization techniques (proximal defense mechanisms such as telling oneself that one is quite healthy and unlikely to die any time soon). Afterward, when the activated knowledge has been suppressed, they show a number of defense mechanisms, which have collectively been called cultural worldview defense (CWD), serving as distal defense mechanisms to ensure “symbolic immortality” (e.g., Greenberg et al., 1986; Pyszczynski et al., 1999). Examples of CWD are in group biases (Castano et al., 2002), nationalistic biases (Nelson et al., 1997), and negative reactions to those who infringe cultural values (Rosenblatt et al., 1989).

In line with the assumption of the suppressive effects of proximal defense mechanisms, numerous studies have not found influences of existential threat on affect self-reports (see also Burke et al., 2010, for review) despite consistent effects of mortality reminders on CWD (see also Burke et al., 2010). TMT literature therefore usually differentiates between the potential for existential anxiety, which increases after mortality reminders and drives proximal and distal defense mechanisms, and actual experienced existential anxiety, which usually does not change after mortality reminders (Greenberg et al., 2003). The authors propose that indirect measures of affect that circumvent subjective self-reports might be able to pick up on this increased potential for anxiety in the absence of changes in self-reported affect.

In the present study, the authors investigated whether a death reminder leads to changes in implicit negative affect in the absence of changes in explicit negative affect. As a death reminder, the authors used a reminder of Islamic terrorism (see Pyszczynski et al., 2003b; Das et al., 2009). It can be assumed that the threats of terrorism, in particular, Islamic terrorism, are particularly salient to soldiers waiting to be deployed to an Islamic country with frequent terrorist attacks. The authors thus drew on a sample of German soldiers that were soon to be deployed to Afghanistan or Kosovo while several German soldiers had been wounded in action in Afghanistan during the preceding months. In addition, several German soldiers had been killed in action the previous year (Tagesschau, 2013). As the soldiers under investigation have enlisted to military duty, explicit processing of negative affect, which typically considers a withdrawal behavior, is unrewarding. Therefore, it is likely that negative affect is not reported or suppressed, leading to absent self-reported negative affect changes. Even so, the authors expect that terror reminders show increased implicit negative affect and potentially decreased implicit positive affect after terror reminders. The authors do not expect to see such effects in the control condition.

## Methods

### Materials

#### IPANAT

The version of the IPANAT used in this study consisted of six artificial words (Quirin et al., 2009) that were rated on four-point scales anchored at 1 (*does not fit at all*) and 4 (*fits very well*) according to how much they sounded like six adjectives (three positive and three negative adjectives). The positive adjectives were *gelassen* [calm], *entspannt* [relaxed], and *umsichtig* [mindful/observant] while the negative adjectives were *traurig* [sad], *bedrückt* [depressed], and *unbehaglich* [uneasy]. To compute positive and negative implicit affect scores, the authors first averaged scores for each adjective across all artificial words. The authors then computed grand means across the three positive and the three negative adjective scores, respectively (cf. Quirin et al., 2009). Cronbach's alpha was 0.78 for the three positive adjectives and 0.79 for the three negative adjectives.

#### Explicit Affect Scale

The explicit affect scale consisted of the same adjectives as the ones used in the IPANAT. Participants rated on four-point scales ranging from 0 (*not at all*) to 3 (*very much*) how much each of the adjectives described their current affective state. Cronbach's alpha was 0.40 for the three positive adjectives and 0.83 for the three negative adjectives. Although the internal consistency of the explicit positive affect scale was unsatisfactory, the authors still used the mean scores across all three adjectives in the analyses presented in this paper in order to maintain the equivalence of the implicit and explicit scales. Not least, the authors did so because preliminary analyses showed that the significance of the effects did not substantially change.

### Participants

A total of 55 German soldiers (53 male, 2 female; *M*_age_ = 23.11, *SD*_age_ = 1.54) studying at Helmut Schmidt University, an educational institution of the German armed forces located in Hamburg, Germany, participated voluntarily in the study. Participants majored in various subjects (industrial engineering: *n* = 18, business administration: *n* = 9, electrical engineering: *n* = 8, mechanical engineering: *n* = 6, other majors: *n* = 11). All participants were to be deployed to Afghanistan or Kosovo in the near future. The authors used the G^*^Power 3 program of Faul et al. (2007) to conduct a sensitivity power analysis with the following parameters: for the independent *t*-test, two-tailed, α error probability = 0.05, *N* = 52 (26 for each group), and Power = 0.81. This analysis showed that the analysis is sensitive enough for effects size *r* ≥ 0.37. This expected effect size is also in accordance with a meta-analysis on Terror management theory that results in a substantial effect size (Burke et al., 2010).

### Procedure

Participants were tested in a group session in a lecture hall at Helmut Schmidt University. At the beginning of the session, the participants were randomly assigned to either a terrorist group (*N* = 27) or a TV control (*N* = 28) and were asked to complete a memory-perceptual task, which served as a cover story of the experimental manipulation. More specifically, the terrorist group was first instructed to subsequently recall the terrorist attacks in New York (September 11th, 2001), Madrid, and London, as well as the thwarted terrorist attacks in Germany. The participants in this group were then shown photographs of the terrorist attacks and read a corresponding short report. The pictures were arranged from left to right in a way that the location of the attacks approached their home country, Germany, both spatially and temporally (New York/USA, 9/11/2001, Madrid/Spain 03/11/2004, London/United Kingdom 07/07/2005, Koblenz/Germany 07/31/2006). After viewing these pictures and reading the report, the participants were asked to write down experiences and thoughts associated with the attacks. By contrast, the control group was instructed to think of a typical TV evening and was shown four pictures related to watching TV (e.g., the logo of a popular German news broadcast). Afterward, this group of participants was asked to write down experiences associated with an ordinary TV evening for an equal amount of time as the experimental group.

After these inductions, the IPANAT was administered to assess implicit positive and negative affect, followed by the explicit self-report affect measure. The authors kept constant the order of implicit and explicit affect measures because (a) the authors were mainly interested in implicit affect changes, and therefore, eliminated the chances that the self-report measure influences the implicit one as it has been previously found that explicit measures influence ratings on implicit measures (Bosson et al., 2000); (b) the authors expected the affect changes to be greatest immediately after the terrorism reminder. Next, the participants completed a task irrelevant to the current research question and finally were thanked for their participation.

## Results

Means and standard deviations for implicit and explicit affect in the two experimental conditions (terrorism vs. TV) can be seen in Table 1. *T*-tests comparing positive and negative explicit affect between the two conditions yielded no significant effects either for explicit positive affect, *t*_(53)_ = 0.711, *p* = 0.48, *r* = 0.10 or for explicit negative affect, *t*_(53)_ = −0.938, *p* = 0.35, *r* = −0.13. Notably, examination of these conditions means in Table 1 shows that these non-significant differences point in the opposite direction as would be expected from the death-anxiety (or death-negative-affect) connection, since positive affect was slightly higher and negative affect slightly lower in the terror compared to the TV condition.

**Table 1:** Descriptive statistics of dependent variables as a function of experimental condition.

	**Terrorism (*****N*** **=** **27)**			**TV (*****N*** **=** **28)**		
	***M***	***SD***	**95% CI**	***M***	***SD***	**95% CI**
**Implicit**						
Positive affect	**1.94**	0.49	[1.77; 2.12]	**2.30**	0.25	[2.21; 2.39]
Negative affect	**2.38**	0.44	[2.21; 2.54]	**1.91**	0.41	[1.76; 2.07]
**Explicit**						
Positive affect	1.63	0.68	[1.37; 1.87]	1.51	0.55	[1.29; 1.71]
Negative affect	0.52	0.55	[0.32; 0.74]	0.69	0.79	[0.42; 1.02]

*Means shown on bold type within a row differed significantly at p < 0.001*.

Analogous *t*-tests for implicit positive and negative affect indicated significantly lower values of implicit positive affect and significantly higher values of implicit negative affect in the terror compared to the TV condition (see Table 1), *t*_(53)_ = −3.45, *r* = 0.43, *p* = 0.001 for implicit positive affect and *t*_(53)_ = 4.10, *r* = −0.49, *p* < 0.001 for implicit negative affect. Degrees of freedom of the *t*-test for positive affect have been adjusted to account for non-equal variances as indicated by a significant Levene's test.

## Discussion

The authors investigated implicit and explicit affective reactions of German soldiers waiting to be deployed to a country, where terrorist attacks were daily fare (Afghanistan) or likely (Kosovo). The authors presented the participants in this study with past and current reminders of Islamic terrorism in the United States and Europe (vs. a control TV condition). The authors expected affective changes at the implicit affect level, using the IPANAT. The findings of this paper corroborated the hypotheses: the authors found that the predicted increase in implicit negative and decrease in implicit positive affect in the terrorism condition relative to the TV control and the self-report measure taken after the manipulation (after the indirect measure in which the very same adjectives were rated) did not show an impact of the manipulation.

The authors drew on a sample of soldiers who were about to be deployed, and the soldiers did not report negative affect-related responses. Negative affect typically signals withdrawal tendencies, but the soldiers cannot reveal them, as they have contracted a professional obligation, let alone the possible implicit peer-group pressure. This is the case, as the authors indirectly captured the higher negative affect for the soldiers *via* the IPANAT. Therefore, findings of this paper do not contradict the research that found self-reported negative affect in response to terror reminders in other samples that differ from the samples analyzed in this paper in their tendency to report their negative affect (e.g., Bleich et al., 2009).

It is worth noting that the authors found the effects of our manipulation on both implicit positive and implicit negative affect. This suggests that the terror reminder may not only have induced negative affect but also reduced positive affect (e.g., paralysis or reduced cheerfulness) in the face of previous or potential future terror. In this context, it needs to be mentioned that for our sample of participants, the terrorism induction was personally relevant to their momentary life situation and not only hypothetical, as in many studies dealing with existential threats in the context of mortality salience research. Because of that, it is important to replicate these implicit affect findings in samples from different populations.

Given that many non-Muslim citizens of Western countries may associate Islam (or some form of Islam) with terrorism and their death, future studies might also explore if priming the Islam-related stimuli activate terror management processes in non-Muslim soldiers and/or civilians. If so, future studies should address the question of whether this association is related to explicitly expressed or implicit anti-Muslim prejudice.

The authors want to critically object that terror reminders may also elicit anger (Lambert et al., 2010), an approach-related negative emotion. However, the authors did not measure anger here. It should be noted that the goal of the present study was to investigate whether soldiers might show the implicit negative affect (related to withdrawal motivation), which corresponds to the implicit negative affect detected. This hypothesis was corroborated and thus suggested the usefulness of the IPANAT in capturing implicit affect.

Based on the previous studies using the IPANAT, the authors presented the implicit affect measure (IPANAT) first and the explicit affect measure afterward (see Quirin et al., 2009). However, affective phenomena are dynamic, implicating that changes in them are transient (see e.g., Verduyn et al., 2009). Thus, it would be desirable to reverse the order of presentation of the implicit and explicit affect measures in future studies to see if the present findings replicate. Moreover, an important question is how implicit and explicit change relative to one another over time, in general, or after a mortality reminder. Notice that the authors measured affect during the proximal defense phase when participants might have engaged in active denial (cf. Pyszczynski et al., 1999). However, both implicit and explicit affect might likely return to the baseline after the suppression of mortality awareness. Future studies should investigate this by measuring both implicit and explicit affect immediately after a mortality reminder.

In conclusion, the current study provides further evidence for the validity of the IPANAT as a measure of affective phenomena in general and as a measure that has the potential to assess affect indirectly. We have demonstrated that the IPANAT captures a type of existential threat which has theoretically been connected to affective phenomena, and which can influence both implicit negative and positive affect.

## Data Availability Statement

The raw data supporting the conclusions of this article will be made available by the authors, without undue reservation.

## Ethics Statement

The studies involving human participants were reviewed and approved by Ethics Board, PFH Göttingen. The patients/participants provided their written informed consent to participate in this study. Written informed consent was obtained from the individual(s) for the publication of any potentially identifiable images or data included in this article.

## Author Contributions

MQ, MK, and UL conceptualized the study design. UL and FM analyzed the data. MQ, MK, FM, and HK wrote the draft of the manuscript. All authors contributed to the article and approved the submitted version.

## Conflict of Interest

The authors declare that the research was conducted in the absence of any commercial or financial relationships that could be construed as a potential conflict of interest.
